# Does actually mean chromosome number increase with latitude in vascular plants? An answer from the comparison of Italian, Slovak and Polish floras

**DOI:** 10.3897/CompCytogen.v6i4.3955

**Published:** 2012-11-19

**Authors:** Lorenzo Peruzzi, Grzegorz Góralski, Andrzej J. Joachimiak, Gianni Bedini

**Affiliations:** 1 Department of Biology, Botany Unit, University of Pisa, via L. Ghini 5, 56126, Pisa, Italy; 2 Department of Plant Cytology and Embryology, Jagiellonian University, Grodzka 52, 31-044 Cracow, Poland

**Keywords:** Biogeography, chromosome number, cytogeography, cytotaxonomy, Europe, polyploidy

## Abstract

We compared chromosome number (CN) variation among vascular floras of three different countries with increasing latitude in the Boreal hemisphere: Italy, Slovakia, Poland. Aim of the study was to verify whether the patterns of CN variation parallel the differences in latitudinal ranges. The three datasets comprised 3426 (Italy), 3493 (Slovakia) and 1870 (Poland) distinct cytotypes. Standard statistics (ANOVA, Kruskal–Wallis tests) evidenced significant differences among the three countries, mean CN increasing together with latitude. On the contrary, an inverse relation (r = -1) was evidenced among the frequency of odd CNs and latitude. Our results show that the hypothesis of a polyploid increase proportional with distance from the Equator seems to be confirmed, when territories from the same hemisphere are compared.

## Introduction

Chromosome number is the most basic feature concerning the genome of a species, and it is also the easiest to obtain, technically. For this reason, since 1882 (Garbari et al. 2012), chromosome number data for many plant organisms have been accumulated worldwide accounting for about one third of plants being now known in this respect (Stace 2000). Although cytotaxonomy had become less popular in the end of twentieth century (Guerra 2012), in the last years, a growing interest of scientific botanical community was devoted to plant chromosome number databases (Stuessy 2009), especially those in digitized format (Gacek et al. 2011; Bedini et al. 2012a, c).

As already pointed out by Peruzzi et al. (2011) and Bedini et al. (2012a, b, c), plant chromosome number databases are a useful tool for systematic comparisons of geographical or taxonomical groups of plants. In these studies, profound differences in chromosome number variation were evidenced for instance between Italian and antipodean New Zealand vascular flora, at various taxonomical scales (vascular plants as a whole, single orders), suggesting also possible different evolutionary dynamics among the two hemispheres (Peruzzi et al. 2011). Also just within Italian flora, a significant increase in mean chromosome number was evidenced to follow a bioclimatic/latitudinal gradient (Islands→southern peninsular Italy→northern Italy) (Bedini et al. 2012a) and specific orders and families where shown to be marked by peculiar chromosome number variation patterns (Bedini et al. 2012b).

A natural prosecution of the above mentioned studies, concerning geographical variation of mean chromosome number, was to extend the sample coverage, by selecting further countries (from the same hemisphere) to test the hypothesis that mean chromosome number in vascular plants tends to increase in parallel with latitude / cooler bioclimate. Accordingly, the aim of this study is to quantitatively evaluate chromosome number variation of vascular floras among three countries with increasing latitude and decreasing altitudinal range (Table 1): Italy, Slovakia and Poland.

**Table 1.** Range of latitudes (in degrees and in km) and altitudes for the considered countries.

	**Degrees**	**Km**	**Altitude**
Italy	35°29' to 47°05'N	1500	0–4810 m a.s.l.
Slovakia	47°40' to 49°35'N	200	94–2655 m a.s.l.
Poland	48°59' to 54°49'N	650	-2–2499 m a.s.l.

## Methods

### Data source

Chromosome numbers from the considered countries were taken from available online databases. *Chrobase.it* (Bedini et al. 2010 onwards) stores the available karyological information about Italian vascular flora, in terms of chromosome number (2nand/or n) and B-chromosome occurrence, along with main geographic-administrative data and literature references (Bedini et al. 2012a). The “Karyological database of ferns and flowering plants of Slovakia” (www.chromosomes.sav.sk/ ) stores the available karyological information about Slovak vascular flora, and was recently published also as hard-print book (Marhold et al. 2007). Finally “Chromosome number database – PLANTS” (Góralski et al. 2009 onwards) stores the available karyological information about Polish angiosperms. The latter database was also integrated by a recent survey on Polish ferns (Ivanova and Piekos-Mirkova 2003). The total number of cytotypes retained for each dataset (ITA: Italy; SK: Slovakia; PL: Poland) was obtained by excluding counts in multiple copy (i.e. the same chromosome number for the same species). Eventual n counts (a minority in the three datasets) were transformed to 2n. Italian dataset coverage is about 35% of vascular plants (Bedini et al. 2012a), the Slovak dataset about 60% (Marhold et al. 2007), and the Polish one about 40% (Gacek et al. 2011). The families circumscription followed APG III (2009), Chase and Reveal (2011) and Christenhusz et al. (2011a–b).

### Data analysis

Similarly to Bedini et al. (2012a, b), the following data were calculated for each dataset: mean chromosome number (CN hereafter), median, mode, Coefficient of Variation of CN (CV_CN_), frequency of B-chromosomes occurrence (*f*B), frequency of odd CN (*f*OCN), not considering B-chromosomes. ANOVA was used to test statistical differences in CN among considered groups. If ANOVA was not applicable (Levene test), then the non-parametric U Mann-Whitney / H Kruskal-Wallis test was used.

## Results

**Figure 1.**
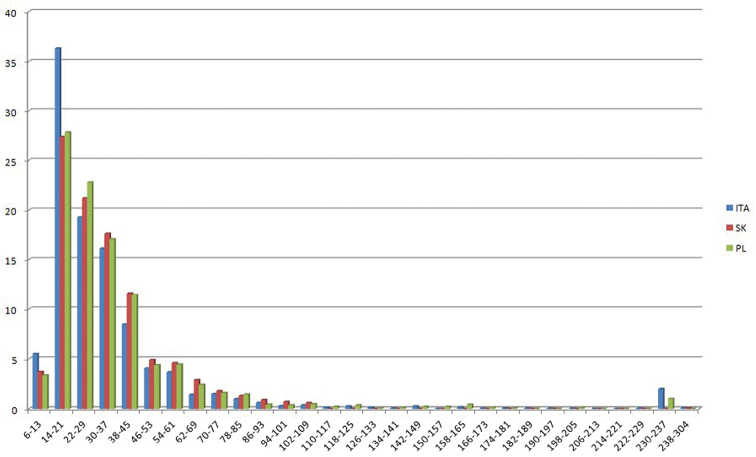
Histograms showing the percentage frequencies (y-axis) of 2n chromosome numbers, grouped in classes (x-axis) known for the Italian (ITA), Slovak (SK) and Polish (PL) vascular flora.

A total of 146 different CNs were found, ranging from 2n = 6 (in all datasets) to 2n = 304 (in the Slovak dataset only). The families included in the datasets were 107 for Italy, 123 for Slovakia and 114 for Poland. Of them, 82 were shared by all datasets. Most of the data (39–40% of each dataset) were concentrated in five families: Asteraceae, Brassicaceae, Fabaceae, Poaceae, Ranunculaceae (Table 2). CNs are apparently distributed in different proportions in the three geographical areas (Table 3; Figure 1). The most frequent (modal) CN in Italy is 2n = 18; in Slovakia it is 2n = 16 and in Poland 2n = 28. Despite this, mean CN is increasing from Italy, through Slovakia, to Poland (Table 1). This difference is supported by ANOVA (F = 22.412, p < 0.000), despite the absence of a significant distinction between Slovakia and Poland. On the contrary, the frequency of odd CNs (*f*OCN) tends to decrease from Italy to Poland (Spearman correlation between mean CN and *f*OCN: r = - 1.0, p < 0.01), while the frequency of B-chromosomes is nearly 8-fold more frequent in Italy than in the other two countries. Indeed, B-chromosomes occur in 246 registered cytotypes (148 taxa) of the Italian vascular flora, in 65 cytotypes (27 taxa) of the Slovak flora and in 39 cytotypes (19 taxa) of Poland flora. Among the taxa showing B-chromosomes, their mean number is 2.03 ± 1.75 in Italy, 2.80 ± 1.99 in Slovakia and 1.95 ± 1.07 in Poland. Since the data on B-chromosome numbers did not follow a normal distribution, we performed the non-parametric Kruskal–Wallis test, which failed, however, to find significant differences between the number of B-chromosomes among the three geographical areas.

**Table 2.** Most represented families in the three datasets (> 100 registered cytotypes in at least one country).

	**Italy**		**Slovakia**		**Poland**	
	**cytotypes**	**%**	**cytotypes**	**%**	**cytotypes**	**%**
Amaryllidaceae	118	3.4	58	1.7	24	1.3
Asteraceae	579	16.9	573	16.4	275	14.7
Asparagaceae	135	3.9	53	1.5	21	1.1
Brassicaceae	193	5.6	238	6.8	80	4.3
Caryophyllaceae	133	3.9	145	4.2	56	3.0
Cyperaceae	56	1.6	110	3.1	36	1.9
Fabaceae	306	8.9	180	5.2	81	4.3
Juncaceae	9	0.3	103	2.9	18	1.0
Lamiaceae	111	3.2	127	3.6	62	3.3
Orchidaceae	158	4.6	62	1.8	30	1.6
Plumbaginaceae	128	3.7	4	0.1	1	0.1
Poaceae	166	4.8	251	7.2	209	11.2
Ranunculaceae	144	4.2	152	4.4	91	4.9
Rosaceae	27	0.8	187	5.4	118	6.3
other families	1163	33.9	1250	35.8	768	41.1

**Table 3.** Chromosome number parameters calculated for each country dataset.

	**N°cytotypes**	**CN**	**± SD**	**median**	**mode**	**C_VC_N**	**fB**	**fOCN**
Italy	3426	30.560	22.060	24	18	72.186	0.071	0.087
Slovakia	3493	33.818	12.728	28	16	37.637	0.019	0.070
Poland	1870	33.820	23.386	28	28	69.149	0.021	0.044

## Discussion

According to our results, it was possible to confirm that, in Boreal hemisphere, mean CN in vascular plants tends to increase with increasing latitude, as already suggested by Bedini et al. (2012a) concerning Italy. Median and modal CN are less variable and not very useful to assess relationships among territories. Especially mode seems prone to reflect a casual abundance of certain taxa in the datasets. Indeed, Slovak and Polish floras, otherwise not statistically distinct, shows modal CNs 2n = 16 and 2n = 28, respectively. This is due to a number (62) of diploid Brassicaceae with *x* = 8 counted in the former country, and a number (56) of tetraploid *Rubus* Linnaeus, 1753 with x = 7 counted in the latter. The scarce differentiation between Slovakia and Poland could be easily explained by their shared administrative borders, with partial overlap of latitude range (cfr. Table 1). On the other hand, a possible influence of altitudinal range - in shaping CN variation among our datasets - cannot be ruled out too, since this parameter shows an exactly inverted variation trend respect with latitude variation (Table 1).

The idea that polyploidy tends to increase with latitude is not new (Löve and Löve 1957, Hanelt 1966, Hair 1966, Stebbins 1971, Levin 2002), but ploidy levels are not easy to assess on large datasets, with coverage comparable to that of current (either online or hard-printed) CN databases and atlases.

The use of mean CN as a proxy of polyploidy has the advantage to be easier to assess and more objective, albeit less precise. Indeed, CNs are unquestionable, while basic CNs are often subjective (see for instance the recent debate in Cusimano et al. 2012). Also the ancestral CN reconstructions are currently based on probabilistic models (Mayrose et al. 2010).

A further interesting point to address with further research is the seemingly different pattern of CN variation among the two hemispheres: Peruzzi et al. (2011) evidenced striking differences among Italy and New Zealand, two nearly antipodean countries. This could be due, to a certain degree, to the fully insular nature of the latter territory, where mean CN is about 2-fold. In order to positively verify whether the CN evolution dynamics in the Austral hemisphere are comparable to those in the Boreal one or not, it could be useful to compare different territories with increasing latitudes, for instance in the southern parts of America and Africa. Unfortunately, as far as we are aware, CN databases covering those territories are not available, or not significant in coverage of flora. Indeed, very recently an online cytogenetic database of Chilean plants was made available (Jara-Seguel and Urrutia 2011 onwards), but only 2.8% of Chilean angiosperm flora was karyologically studied (Jara-Seguel and Urrutia 2012). Similar degree of coverage exists for plants from Paraguay (Molero et al. 2001). Of course, the use of territories circumscribed by ecological and/or biogeographical criteria, instead of countries, could be even more useful to address these questions. Unfortunately, such kind of CN databases do not exist.

Contrary to what was observed for Italy (Bedini et al. 2012a), the frequency of B-chromosomes (*f*B) does not follow a geographical gradient, but in all the three considered countries values were higher than those reported for New Zealand (Peruzzi et al. 2011). Indeed, the adaptive/ecological role of B-chromosomes is still a controversial issue (Jones 2012). Concerning the frequency of odd CNs (*f*OCN), it is clearly decreasing with increasing latitude, while New Zealand has a value intermediate between Slovakia and Poland (Peruzzi et al. 2011). Maybe the latter finding could be related with a different frequency of apomictic and/or holocentric species in the considered territories.
